# Epidemiology of gastric cancer in Africa: a systematic review and meta-analysis protocol

**DOI:** 10.1186/s13643-019-1214-2

**Published:** 2019-11-13

**Authors:** Celestin Danwang, Jean Joel Bigna

**Affiliations:** 1 Epidemiology and Biostatistics Unit, Institute of Experimental and Clinical Research, Université Catholique de Louvain, Brussels, Belgium; 2 Department of Surgery and Specialties, Faculty of Medicine and Biomedical Sciences, University of Yaoundé 1, Yaoundé, Cameroon; 3 Department of Epidemiology and Public Health, Centre Pasteur of Cameroon, Yaoundé, Cameroon; 4 School of Public Health, Faculty of Medicine, University of Paris Sud XI, Le Kremlin-Bicêtre, France

**Keywords:** Epidemiology, Gastric cancer, Africa, Review

## Abstract

**Background:**

Gastric cancer is actually known as the sixth most frequent cancer and the second cancer-related cause of death worldwide. If studies giving an overview of current epidemiology of gastric cancer in Europe, Asia, and the USA are available, in Africa, studies reporting recent data on gastric cancer are sparse. This systematic review and meta-analysis aim therefore to provide relevant data on contemporary epidemiology of gastric cancer in Africa in terms of prevalence, incidence, and case fatality rate.

**Methods and design:**

We will include cohort, case-control, cross-sectional studies, and case series with more than 30 participants. EMBASE, PubMed, Africa Index Medicus, Africa Journals Online, and Web of Science will be searched for relevant abstracts of studies published and unpublished between January 1, 2000, and April 30, 2019, without language restriction. The review will be reported according to the MOOSE (Meta-analysis Of Observational Studies in Epidemiology) guideline. After screening of abstracts, study selection, data extraction, and risk of bias assessment, we shall assess the studies individually for clinical and statistical heterogeneity. Random-effect meta-analysis will be used to pool studies judged to be clinically homogenous. The Egger test and visual inspection of funnel plots will be used to assess publication bias.

**Discussion:**

This review will provide relevant data on the current burden of gastric cancer in Africa.

**Systematic review registration:**

PROSPERO CRD42019130348.

## Background

Gastric cancer is the sixth most frequent cancer and the second cancer-related cause of death worldwide [1]. Known as the most prevalent cancer in the USA three decades ago, the incidence of gastric cancer has been steadily decreasing in the USA and worldwide [2–4]. Actually, there is a huge discrepancy in the global distribution of morbidity and mortality related to gastric cancer, with eastern Asia having the highest burden of this disease [2, 3]. Gastric cancer affected disproportionately men and women, with men being three times more affected than women [2, 3, 5]. The global age-standardized incidence rate is about 35.4 for 100,000 in men and 13.8 for 100,000 in women [3]. Despite improvements in the diagnostic and treatment of gastric cancer, it is still associated with high mortality worldwide. The survival rate at 5 years has been 20% in most countries in the world [6, 7].

The declining trend of the global incidence of gastric cancer is related not only to an early diagnosis but also to a better knowledge and control of the risk factors of this pathology. Indeed, the discovery of the role played by *Helicobacter pylori* in the pathogenesis of this disease as well as the influence of dietary factors (salty and smoked food, western pattern diet), low socio-economic level, hygiene, and smoking, has substantially reduced the incidence of this condition in developed countries [4, 5]. Japan, which is between the ten countries with the highest prevalence of gastric cancer in the world, has contributed significantly to a better understanding of the pathogenesis of gastric cancer and the role of early endoscopic screening in the reduction of its prevalence [2, 7]. In this country, a 50% reduction in mortality rate associated with gastric cancer between 1975 and 2005 has been observed [2].

Most of the studies published on the occurrence of gastric cancer in Africa are modeling studies or do not always consider both published and unpublished literature, but cancer registries’ data to make their estimations [8–11]. In many African countries, the reporting system and thus cancer registries have many weaknesses that may lead to an incorrect estimation of the true epidemiology of the disease [12]. An estimation of epidemiology of gastric cancer using a different approach based mainly on available contemporary literature may give a different picture of this condition in the African continent which has an epidemiological peculiarity because of the double burden of infectious diseases (among which *Helicobacter pylori* infections) and non-infectious diseases [13]. We therefore propose the current study with the aim to synthetized data on contemporary epidemiology of gastric cancer in Africa throw a systematic review with meta-analysis in order to provide relevant and accurate data that can help to build efficient and sustainable strategies by policymakers to curb down the burden of this disease.

### Review question

What is the epidemiology of gastric cancer in people living in Africa?

### Objectives

This systematic review and meta-analysis aim at the following:

1. Determine the incidence of gastric cancer in people living in Africa
2. Determine the prevalence of gastric cancer in people living in Africa
3. Determine the case fatality rate of gastric cancer in people living in Africa

## Methods and design

This systematic review and meta-analysis will be reported in conformity with the MOOSE (Meta-analysis Of Observational Studies in Epidemiology) guideline [14]. The Preferred Reporting Items for Systematic Review and Meta-Analysis (PRISMA-P) for Protocol was used to report this protocol [15]. The PRISMA-P checklist is attached as Additional file 1. This protocol is registered in PROSPERO with the number CRD42019130348.

### Criteria for considering studies for the review

#### Population

We will include participants with gastric cancer of all age and gender to determine the case fatality rate. To determine the prevalence and incidence, we will consider general population and populations with specific diseases or conditions.

#### Outcomes

We will consider studies reporting the following outcomes with enough data to compute these estimates: prevalence, incidence, and case fatality rate of gastric cancer. Gastric cancer had to be diagnosed with anatomo-pathological examination; otherwise, the definition used by the author will be reported. We will exclude studies in which relevant data are impossible to extract even after contacting the corresponding author.

The primary outcome will be the prevalence of gastric cancer representing the number of cases divided by the total population in a given time point. The secondary outcomes will be the incidence rate (number of new cases during a period divided by the number of participants’ time of follow-up) and case fatality rate (number of deaths divided by the number of patients with gastric cancer).

#### Types of studies

We will include cohort studies, case-control studies, cross-sectional studies, and case series with more than 30 participants. Letters to the editor, case reports, narrative reviews, commentaries, perspectives, and editorials will be excluded.

### Search strategy for identifying relevant studies

The search strategy will be conducted in the following sections.

#### Bibliographic database searches

Relevant articles published on gastric cancer will be identified by searching EMBASE, PubMed, Africa Index Medicus, Africa Journals Online, and Web of Science between January 1, 2000, and April 30, 2019. Text words and medical subject heading terms related to gastric cancer will be used including “gastric cancer,” “stomach neoplasm,” “gastric neoplasm,” and “stomach cancer.” The name of the African country in the language relevant to this country will also be applied. Additional file 2 shows the full search strategy for PubMed that will be adapted to fit with other databases. No language restriction will be applied. For articles published in a language other than Spanish, English, and French, an experienced translator in the concerned language will be contacted for translation.

#### Searching for other sources.

We will scan the references of all relevant articles for additional data sources missed during our search, and their full texts will be retrieved. References of pertinent reviews will also be scanned. Cancer registries, official governments documents related to health, government reports, and conference papers will also be scrutinized.

### Selection of studies for inclusion in the review

Two reviewers (CD and JJB) will independently evaluate the studies obtained from the searches, using an assessment form to ensure that the selection criteria are reliably applied. These reviewers will screen the titles and abstracts of papers obtained, after which the full texts of potentially eligible papers will be retrieved by at least one reviewer. The two reviewers will independently review the full text of each potentially eligible study, compare their results, and resolve any discrepancy by discussion. For duplicate studies published in more than one report, the one reporting the largest sample size will be considered. Studies with inaccessible full text either online or from the corresponding author will be excluded.

### Assessment of methodological quality and reporting of data

Methodological quality and risk of bias of included studies will be assessed using an adapted version of the risk of bias assessment tool developed by Hoy et al., and reported accordingly [16].

### Data extraction and management

All references identified after implementation of the searched strategy will be imported inside the Endnote software. All records obtained from various databases will be combined in a single Endnote library, and the duplicates will be removed. A data extraction form will thereafter be used to collect information on the surname of the first author, year of publication, country where the study was conducted, study design, study area (rural versus urban), age groups (children, adolescent, adult, elders), sample size, mean or median age, gender, specific characteristics of the study population, histological type of the cancer, location of the tumor, classification of the cancer (according to Union for International Cancer Control classification), prevalence, incidence, and case fatality rate of gastric cancer in the study. For multinational studies, the data will be reported for the individual countries. Where it is impossible to disaggregate data for such studies by country, data will be presented as a single study, and the individual countries which participated in the study will be reported.

### Data synthesis and analysis

We plan to do a meta-analysis after data collection. Unadjusted prevalence and incidence with their standard errors for each study will be recalculated based on the information of crude numerators and denominators provided by individual studies. The variance of the study-specific prevalence will be stabilized with the Freeman-Tukey double arcsine transformation [17], before pooling the data using a random-effect meta-analysis model. All pooled estimates will be reported with their 95% confidence interval. Heterogeneity will be assessed using the *χ*^2^ test on Cochran’s *Q* statistic and quantified by calculating *I*^2^ [18]. Values of 25%, 50%, and, 75% for *I*^2^ will respectively represent low, medium, and high heterogeneity. We will assess the presence of publication bias using funnel plot inspection and Egger’s test if there are three studies or more for a meta-analysis [19]. When there will be enough data, meta-regression and subgroup analyses will be performed to investigate the possible sources of heterogeneity using the WHO African subregion, histological type of cancer, gender, and study quality. In case of substantial clinical heterogeneity, a narrative summary of findings will be done. The inter-rater agreement for study inclusion between investigators will be assessed using Cohen’s κ coefficient [20]. Data analyses will be done using the “meta” package of the statistical software R (version 3.2.2, The R Foundation for statistical computing, Vienna, Austria).

### Presentation and reporting of results

The study selection process will be summarized using a flow diagram. Quantitative data will be presented in tables of individual studies, and in summary tables, and forest plots where appropriate. Data will be reported according to histological type of cancers, gender, age group, countries, and African subregions. The quality scores and risk of bias for each eligible study will be reported accordingly. The strength of the body of evidence will be assessed using the GRADE classification.

## Discussion

The purpose of this study is to provide relevant data on the current burden of gastric cancer in Africa. African countries are afflicted by the double burden of infectious and non-infectious diseases, and epidemiological data on the occurrence of those diseases are therefore needed to target the deadliest ones as a priority in order to reduce the high mortality still observed in many countries of the WHO African region. One limitation to this study may be the quality of primary studies included in the review. Indeed, the method used for the ascertainment and classification of gastric cancer may vary across studies. Given the low availability of CT scan and modern imaging techniques in Africa 20 years ago, some major information needs for the stage classification may be missing. Another limitation may be the low number of published studies on gastric cancer in Africa which may lead to an estimation based mainly on unpublished literature. Nevertheless, this study will be the first systematic review with meta-analysis summarizing published and unpublished data on the occurrence and case fatality rate of gastric cancers in Africa. The final report will be published in a peer-reviewed journal.

### Review status

Preliminary searched.

#### Patient and public involvement

Patients and/or the public were not directly involved in this study.

#### Potential amendments

Any amendment in the review process will be reported for transparency, and the PROSPERO registration document modified to make it available online.

## Supplementary information

**Additional file 1:** PRISMA-P (Preferred Reporting Items for Systematic review and Meta-Analysis Protocols) 2015 checklist: recommended items to address in a systematic review protocol*

**Additional file 2:** Search strategy for PubMed from January 1, 2000 and April 30,2019

## Abbreviations

DALY: Disability-adjusted life year
PRISMA: Preferred Reporting Items for Systematic Review and Meta-Analysis
PROSPERO: International Prospective Register for Systematic Reviews
