# Supplementary material for: Epidemiology of gastric cancer in Africa: a systematic review and meta-analysis protocol
Source: Syst Rev. 2019 Nov 13;8:276. doi: 10.1186/s13643-019-1214-2 (PMC6852794; doi:10.1186/s13643-019-1214-2)
Supplement: Supplementary file 2 — Additional file 2: Search strategy for PubMed from January 1, 2000 and April 30,2019 [file 13643_2019_1214_MOESM2_ESM.pdf]

Supplementary File 2: Search strategy for PubMed from January 1, 2000 and April 30, 2019

Search	Search terms
#1	"Stomach Neoplasms"[Mesh] OR “gastric cancer” OR “gastric neoplasm”
#2	(Africa* OR algeria OR angola OR benin OR botswana OR burkina faso OR burundi OR cameroon OR canary islands OR cape verde OR central african republic OR chad OR comoros OR congo OR democratic republic of congo OR djibouti OR egypt OR equatorial guinea OR eritrea OR ethiopia OR gabon OR gambia OR ghana OR guinea OR guinea bissau OR ivory coast OR cote d ivoire OR jamahiriya OR kenya OR lesotho OR liberia OR libya OR madagascar OR malawi OR mali OR mauritania OR mauritius OR mayotte OR morocco OR mozambique OR namibia OR niger OR nigeria OR principe OR reunion OR rwanda OR sao tome OR senegal OR seychelles OR sierra leone OR somalia OR south africa OR south sudan OR st helena OR sudan OR swaziland OR tanzania OR togo OR tunisia OR uganda OR western sahara OR zaire OR zambia OR zimbabwe OR central africa OR central african OR west africa OR west african OR western) africa OR western african OR east africa OR east african OR eastern africa OR eastern african OR north africa OR north african OR northern africa OR northern african OR south african OR southern africa OR southern african OR sub saharan africa OR sub saharan african OR subsaharan africa OR sub saharan african NOT (guinea pig OR guinea pigs OR aspergillus niger)
#3	#1 AND #2
